# Primate social group sizes exhibit a regular scaling pattern with natural attractors

**DOI:** 10.1098/rsbl.2017.0490

**Published:** 2018-01-17

**Authors:** R. I. M. Dunbar, Padraig Mac Carron, Susanne Shultz

**Affiliations:** 1 Department of Experimental Psychology, University of Oxford, New Richards Building, Old Road Campus, Oxford OX3 7LG, UK; 2 Department of Computer Sciences, Aalto University, Espoo, Finland; 3 School of Biological Sciences, University of Manchester, Oxford Road, Manchester M13 9PL, UK

**Keywords:** optimal group size, clustering, social networks, female cohort, evolutionary rates

## Abstract

Primate groups vary considerably in size across species. Nonetheless, the distribution of mean species group size has a regular scaling pattern with preferred sizes approximating 2.5, 5, 15, 30 and 50 individuals (although strepsirrhines lack the latter two), with a scaling ratio of approximately 2.5 similar to that observed in human social networks. These clusters appear to form distinct social grades that are associated with rapid evolutionary change, presumably in response to intense environmental selection pressures. These findings may have wider implications for other highly social mammal taxa.

## 1. Introduction

Mammals live in a variety of social systems with group sizes that vary, both within and between species, from one to several hundred individuals [1]. While most species have a rather casual form of sociality (temporary aggregations around resources), some live in more stable kinds of groupings (most primates, equids, elephants and delphinids, among others). Stable groups of this kind invariably have a characteristic group structure associated with a typical group size and bonded relationships [2], and many analyses have used mean group size to test evolutionary hypotheses. While group size within a species varies as a function of well-known environmental and demographic processes [3,4], there is no general explanation for why group sizes vary so much between species (although there is a long held assumption that ecology plays a central role [5–7]).

This raises the question as to whether, at the taxon level, primate group sizes consist of a single distribution or several discrete distributions (each with its own optimal value, representing some kind of social grade). The former may be favoured where group sizes are flexible and respond facultatively to extrinsic ecological drivers, as predicted by the socio-ecological model [5–7]. The latter might be favoured if social evolution has followed a stepwise pattern [8], although the fact that primate social evolution is predictable does not necessarily tell us anything about resulting group sizes.

In this paper, we ask whether the distribution of primate groups (indexed by the mean group size for individual species) forms a single parametric distribution or is better described by a set of such distributions centred around different means. A single distribution represents the default null hypothesis: species' group sizes are just a random sample across a unimodal distribution. We need to exclude this to be sure that an explanation in terms of a multiple distribution really is true. A multiple distribution implies that there are structural constraints in realized group sizes, such that there are definable ‘attractors' across the range of potential group sizes. Since a number of studies [9–11] have suggested that primate brain evolution may be more directly related to the size of the female cohort than to total group size, we also ask whether the distribution of the numbers of adult females in a group exhibits any such patterning.

## 2. Material and methods

We collated data on mean social group size for 215 primate species (50 strepsirrhines and 165 haplorhines) representing 68 genera, and mean number of adult females per group for 192 species (37 strepsirrhines and 155 haplorrhines)*.* By ‘group' we refer here to stable social groups (see the electronic supplementary material). The data are provided in electronic supplementary material, Dataset S1.

We apply two different methods to detect natural clustering in the data. First, we use a maximum-likelihood estimator (MLE) approach to find a distribution that best describes the data; we test between a number of unimodal and multimodal distributions, using the Akaike information criterion (AIC) to choose the best model. Then, as a check, we use the Jenks natural breaks algorithm to find the optimal number of clusters that minimizes the variance within clusters. Details for both methods, and the procedure for selecting the optimal number of clusters in each case, are given in the electronic supplementary material.

To evaluate the extent to which phylogenetic effects might explain the patterns in the data we used the *physig* routine in R to calculate Blomberg's *K*. Evolutionary rate changes in group size were detected using the variable rates model implemented in *BayesTraits* [12]. This model partitions the phenotypic variance across the tree into two components, a background rate and a branch-specific scalar relative to the background rate. We use this approach to identify whether rate shifts occur across the tree or are focused on a few phylogenies. If there is a signature for rate shifts across the tree, this is strong evidence that patterns of evolution towards attractors are not simply driven by phylogenetic inertia.

## 3. Results

**Figure 1.**
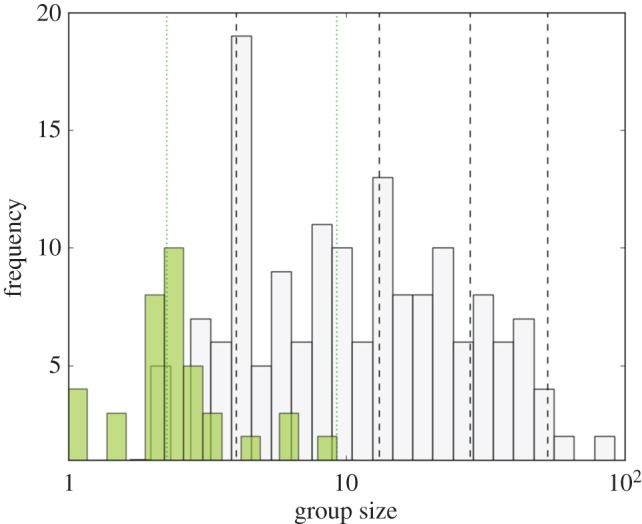
Distribution of mean group size for strepsirrhines (green bars) and haplorhines (grey bars) with the dotted and dashed lines representing the respective mean MLE cluster sizes. (Online version in colour.)

MLE identifies a compound Poisson distribution as by far the most likely of the candidate models (table 1). Both cluster methods identify four clusters as the optimal way to partition the distribution of the 215 species group sizes (electronic supplementary material, figure S1), and are in close agreement on the typical sizes of these clusters (figure 1). MLE gives cluster means at 3.37, 9.91, 24.15 and 52.50, and Jenks finds cluster means at 4.64, 16.31, 31.25 and 53.09 individuals. The average scaling ratio is 2.52 for the MLE series and 2.38 for the Jenks series. We re-ran the analysis on 68 genus means, and obtained virtually the same results: MLE identifies three clusters and Jenks four (see the electronic supplementary material). We also ran the analysis on a dataset of 936 individual group sizes (from [13]), with broadly similar results (see the electronic supplementary material). Finally, we ran separate analyses for strepsirrhines and haplorhines: this yielded similar results, except that strepsirrhines lacked the two largest groupings (see the electronic supplementary material). By contrast, there were no clear patterns in the distribution of female cohort size (see the electronic supplementary material).

**Table 1.** Akaike information criterion (AIC) for how well different distributions describe the pattern of mean species social group size using a maximum-likelihood estimator (MLE) analysis.

distribution	AIC
power law	1657.6
exponential	1458.7
truncated power law	1465.5
Weibull	1457.5
Gaussian	1534.6
lognormal	1449.8
geometrical	1458.7
negative binomial	1488.2
Poisson (single)	3397.1
*compound Poisson (n = 4)*	982.3*

*The italicized value is significantly (*p* < 0.0001) smaller than any of the others, and represents the best fit.

**Figure 2.**
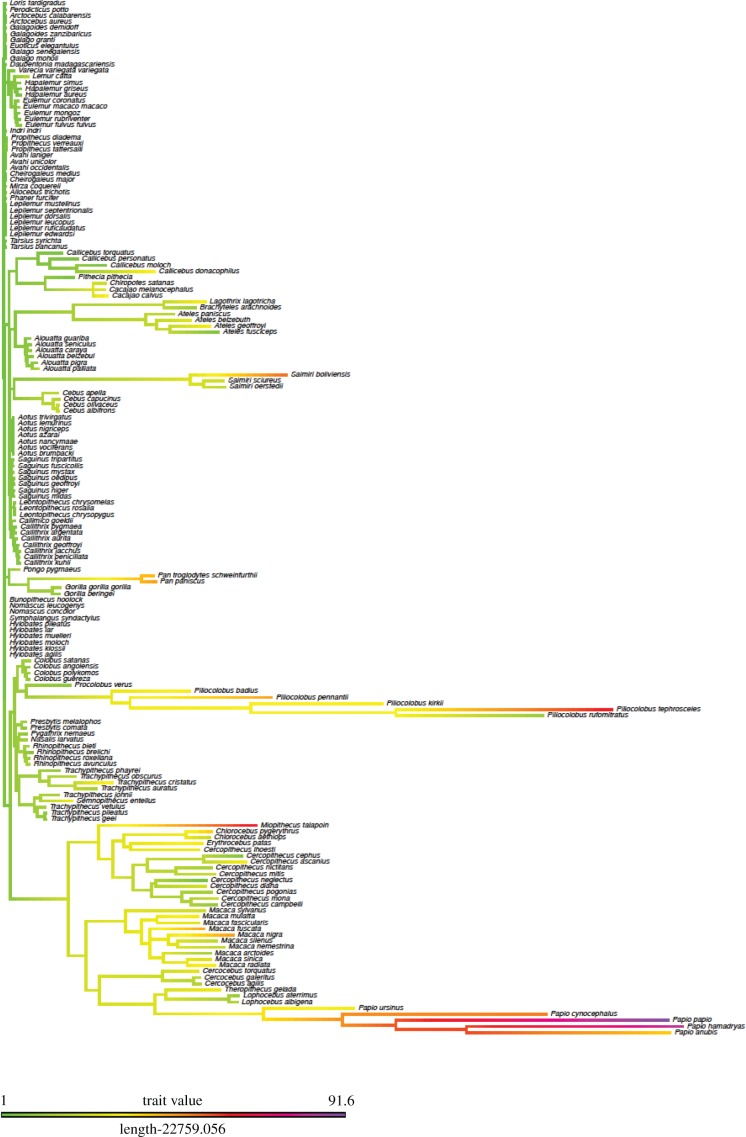
Evolutionary rate changes in social group size across the primate phylogeny. (Online version in colour.)

Kamilar & Cooper [14] reported a very weak phylogenetic signal for group size (Blomberg's *K* = 0.063, *N* = 153 species) and associated demographic variables (*K* < 0.250) in primates. We confirm, with our larger sample and corrected group sizes, a similarly low value at species level (*K* = 0.164). Analysis of the rate changes in group size along lineages (figure 2) reveals that while most species have small groups and show very little change over phylogenetic time, some lineages have undergone unusually rapid changes in group size. This is particularly true of the New World atelines and *Saimiri* (squirrel monkeys), the Old World piliocolobins (red colobus) and cercopithecines (baboons, macaques and guenons), and the genus *Pan* (chimpanzees), with more modest changes among some other haplorhine lineages. These rate changes fall into four natural grades that cut across taxonomic divisions. We identified four main clusters in these rates (electronic supplementary material, Dataset S2). Cross-tabulating these rate clusters with the group size clusters from figure 1 yields a highly significant non-random pattern of association (electronic supplementary material, table S1; *χ*^2^ = 139.7, d.f. = 9, *p* << 0.0001), with a significant positive correlation between the two classifications (Kendall's *τ* = 0.685, *N* = 170, *p* << 0.0001).

## 4. Discussion

The distribution of group sizes across primate species suggests a series of optimal values (attractors) that have a fractal relationship of approximately 2.5. This is close to the scaling ratio of 3 reported for the internal structuring of multilevel societies in both mammals [15] and humans [16–20]. It is the regular fractal pattern in these groupings that should surprise us: there is no obvious reason why groups should vary so consistently in this way if species are completely free to adjust their group sizes to suit their local environments, as implied by classical socio-ecological theory [5–7]. That certain group sizes seem to be attractors, and that these attractors exhibit a very specific fractal pattern, suggests that there may be structural constraints that make some group sizes more stable than others. In this respect, these findings reinforce the claim by Shultz *et al*. [8] that primate societies evolved in a series of stepwise changes. Our results suggest that these phase transitions in structure are associated with corresponding transitions in group size that are probably associated with group structure [21]. These changes, however, appear to be unrelated to the size of the adult female cohort.

Group size seems to have a weak phylogenetic signature compared with most anatomical traits, and neither the clusters nor the rate changes in group size correlate consistently with phylogeny; rather, each cluster is a mixture of taxonomically distantly related lineages, suggesting that group size is likely to be a response to ecological conditions (most likely predation risk [22]). If so, these responses appear to have involved rapid changes, suggestive of strong selection pressures as species occupy a new niche.

It is notable that strepsirrhines and haplorhines differ mainly in the number of clusters they have rather than the mean sizes of these clusters. Moreover, the three strepsirrhine layers (2.3, 6.8 and 15) and three of the four haplorhine layers (5.5, 16.3, 53.1) approximate very closely to the mean sizes of the inner layers widely characteristic of human social networks and organizations (approx. 1.5, approx. 5, approx. 15 and approx. 50) [16–20], with their scaling ratio of approximately 3.0. Note that the 1.5 in the human series does not necessarily refer to romantic partners, but to very close relationships which may be of either or both sexes and may or may not have romantic overtones; it averages approximately 1.5 because some people have one and some two, in about equal proportions [19–20].

What it is about these attractors that makes them so stable is, however, not clear, although evidence from humans suggests that these numbers are unusually stable [23]. The basal cluster of approximately 2.5 individuals in the strepsirrhines clearly reflects the fact that many strepsirrhines are semi-solitary foragers that have nest-sharing by male–female or female–female pairs and trios, with offspring usually ‘parked' in nests prior to dispersal at puberty [24], and hence rarely included in group counts. The basal cluster of approximately 5 in the haplorhine series, and the second cluster in strepsirrhines, can be identified with pairbonded social arrangements (an adult pair plus two or three offspring). This would seem to constitute a minimal functional group. However, note that pairbonded social systems appear to be demographic and evolutionary sinks for primates: once adopted, species seem unable to escape [8], perhaps because major cognitive and behavioural changes (e.g. mate defence) are necessary to support lifelong pairbonds, and these cannot easily be undone or adapted to support other social arrangements [25].

It is worth noting that a grouping of approximately 5 is the standard size of both the inner core of relationships (degree size) in human social networks [26–29] and core grooming networks in primates [30] and this may represent some kind of natural limit on social grouping through direct social contact, irrespective of the form this grouping might take. Thereafter, the fractal pattern suggests that the larger groupings are built up by bolting together several lower level units (i.e. a group of 15 consists of three semi-independent, non-overlapping sub-networks of 5). A scaling ratio close to 2 would suggest that this arises by binary fission [31], whereas a scaling ratio closer to 3 might suggest something more complex that may require more sophisticated cognition to engineer in order to maintain stability over time.

## Supplementary Material

Supplementary Methods and Analyses

## Supplementary Material

ESM Dataset S1: Group Size Data

## Supplementary Material

ESM Dataset S2: Clustering Data

## Data accessibility

The data are provided in the electronic supplementary material.
