# Supplementary material for: Primate social group sizes exhibit a regular scaling pattern with natural attractors
Source: Biol Lett. 2018 Jan 17;14(1):20170490. doi: 10.1098/rsbl.2017.0490 (PMC5803586; doi:10.1098/rsbl.2017.0490)
Supplement: Supplementary Methods and Analyses [file rsbl20170490supp1.docx]

**Primate Social Group Sizes Exhibit a Regular Scaling Pattern**

**with Natural Attractors**

**R.I.M. Dunbar, Padraig Mac Carron and Susanne Shultz**

**ESM**

**Supplementary Methods**

We seek to describe the typical group size characteristic of individual primate groups. Over the past half century, there has been intermittent discussion as to whether “typical” group size is best described by the mean group size or the group size experienced by the average individual (originally by Jarman 1974; more recently by Jovani et al. 2008; Jovani & Mavor 2011). Mean group size is calculated as the average number of individuals found in a group (i.e. each censussed group is counted only once), wheres average experienced group size is the *weighted* average group size (the size of group in which *each* individual is found is counted, such that a group of 50 individuals contributes 50 cases to the calculated average). For obvious reasons, these are not necessarily always the same, especially if the distribution of group sizes is highly skewed and the range very large (e.g. ~120,000 individuals in some seabird colonies: see Jovani et al. 2008). Although the distribution of primate group sizes, both within and across species, is invariably right-skewed, the range of group sizes in primates is, by comparison with flocking birds and herd-forming mammals, very modest indeed: mean species group size in our sample varies between 1-92, and within species varies in the most extreme case only between 10-247 (*Papio papio*). While we recognise the issues raised by this discussion, we note that the claim for experienced group size has largely failed to convince behavioural ecologists to adopt it as a convention. In any case, it seems unlikely that the range of group sizes in which primates are found raises sufficiently serious issues to necessitate a more complex analysis beyond simple overall mean group size. More importantly in the present case, we are here concerned with the number of individuals that live together as an ecological unit (i.e. the mean group size), not with the average individual’s *social* experience. We therefore use conventional mean group size throughout our analyses.

For present purposes, we define a social group as a set of animals that share a ranging area and have some degree of compositional stability through time (allowing for births and deaths). We refer to this as the social group size for the species, and distinguish it from the ecological group size (the set of individuals that share a common ranging area), the foraging group size (the subset of individuals that happen to forage together on any given day) or the reproductive group size (the subset of individuals that form a reproductive unit, as in the case of the harems of gelada and hamadryas baboons). In many primate species, these various types of group are one and the same. In such cases, groups are obvious and fieldworkers have no difficulty in identifying and counting them.

The more difficult cases are represented by those species that have fission-fusion forms of sociality where the foraging group is a subset (or even a super-set) of the social group. Species that have this form of sociality include (1) some nocturnal prosimians, spider monkeys (genus *Ateles*), orangutans and chimpanzees, which have a form of ‘atomistic’ fission-fusion sociality (animals join and leave foraging or sleeping groups as individuals) and (2) certain Asian colobines (notably the genus *Nasalis*) and two species of baboons (*Papio hamadryas* and *Theropithecus gelada*) that have a form of ‘molecular’ fission-fusion sociality (animals live in stable reproductive units, or harems, which associate with each other to varying degrees over time) (Aurelli et al. 2008). In these cases, we use (1) nest group size for the semi-solitary prosimians, (2) community size for spider monkeys and great apes, and (3) the band (in the case of gelada, the team) formed by harems that associate regularly together for *Nasalis* and the baboons (in each case, as defined by the fieldworkers who have studied these species).

We have excluded only the two species of *Mandrillus* from this analysis because the exact form of their sociality remains unclear and subject to debate: they appear to form very large foraging aggregations (Abernethy et al. 2002; Hongo 2014). It is crucial not to confuse temporary aggregations with more permanent social groupings.

Note that our central focus is on the grouping patterns of adult females; we do not, therefore, include all-male groups in our sample, since these are often unstable and typically much smaller than the groups in which females are found, and are perhaps more akin to the kinds of aggregations found in herd- and flock-forming mammals and birds.

We collated data on mean social group size for 215 primate species (50 strepsirrhines and 165 haplorhines) representing 68 genera from Campbell *et al*. (2007), the most recent comprehensive compilation, supplemented, where more recent compilations are available or Campbell et al. provide no data, by Bettridge et al. (2010) for *Papio*, MacCarron et al. (2016) for *Theropithecus*, Lehmann et al. (2008) for African great apes, Dunbar et al. (submitted) for hylobatids, Kappeler (1997) for all lemurs, and Smuts et al. (1997) and DeCasien et al. (2017) for all other genera. We note that some of the data in DeCasien et al. (2017) are unreliable, and we use this source as a last resort only when more reliable sources are not available.

For all semi-solitary lemuriformes, we use nest group size, as specified by Kappeler (1997) and Smuts et al. (1997). For *Pongo*, we use the mean size of the community of residents given by the two field studies (MacKinnon 1974; Singleton & van Schaik 2001) that provide estimates for this. Many comparative analyses use foraging group size (typically 1-2 animals) for *Pongo*, but this is obviously completely inappropriate when comparing against social (as opposed to ecological or foraging) group size for all other species. The value we use (14.0) is based on two studies that give data on community size. It is almost exactly what is predicted by the species’ neocortex size (Dunbar 2011). In this respect, it can reasonably be argued that orang-utans represent the limiting case for the kind of fission-fusion sociality found in great apes, namely that when foraging party size is reduced to one (Lehmann et al. 2010).

We also collated data on the mean number of adult females per group for 192 species (37 strepsirrhines and 155 haplorrhines) from the same sources. For the sex composition data, we required that at least three groups should have been censussed so as to avoid biased sampling since, in most cases, only a small subset of study groups provide data on group composition. The data are provided in *ESM Dataset S1*).

We apply two different methods to detect natural clustering in the data. First, we use the maximum-likelihood estimator (MLE) approach to find a distribution that best describes the data (Clauset et al. 2009). Because the data may not simply be right-skewed but instead composed of a set of independent distributions, we also test a compound Poisson distribution (i.e. where the data are described by a set of Poisson distributions). We chose compound Poisson rather than compound negative binomial because this reduces the number of parameters that have to be estimated (each Poisson distribution has only a single parameter). To identify the parameters for the different distributions, we numerically maximise the log of the likelihood of each distribution using the *optimize* module in Python’s *scipy* (v0.17.1) library. We use AIC to choose the best candidate model. In the case of the compound Poisson distributions, we treat the data as being made up of 1 to *n* Poisson distributions, and calculate the AIC for each value of *n* stopping when it reaches a local minimum.

We then use the Jenks natural breaks algorithm (Jenks 1967) on the group size data to confirm that the MLE findings are robust. The Jenks algorithm is similar to *k*-means clustering but is optimal for one-dimensional data. It partitions the data into successively more clusters based on natural breaks in the distribution in order to find the cluster number that minimises the variance within clusters. Since the goodness of fit for clusters will, in the limit, reach 1.0 only when the number of clusters is the same as the sample size, we follow Coulson (1987) and search for the number of clusters that has a goodness of fit of 0.85; this value is then identified as the optimal number of clusters.

We have used these clustering algorithms (including *k*-means clustering) to search for fractal patterns in a variety of group size data sets, including social networks based on spatial proximity in gelada baboons (*Theropithecus gelada*) (MacCarron & Dunbar 2016), troop size in *Papio* baboons (Dunbar et al., under review), as well as humans social network size in telephone calling datasets (MacCarron et al. 2016) and online environments (Dunbar et al. 2015), the sizes of residential camp sites (Kordsmeyer etal. 2017) and the sizes of historical communities (Dunbar & Sosis 2017). They all generally agree closely with each other.

**Optimal Number of Clusters**

Fig. S1 plots the GVF index of goodness of fit for the Jenks natural breaks algorithm. This index varies between 0 and 1, and gives a measure of the proportion of variance explained when the data are partitioned into N clusters (where N can vary from 2 to ∞). Coulson (1987) recommends a cut-off at GVF=0.85 to identify the optimal number of clusters. In this case, that point (identified by the red dot in Fig. S1) is N=4 clusters. This demarcates the inflexion point where GVF begins to reach an asymptotic value while at the same time minimising the number of clusters.

**
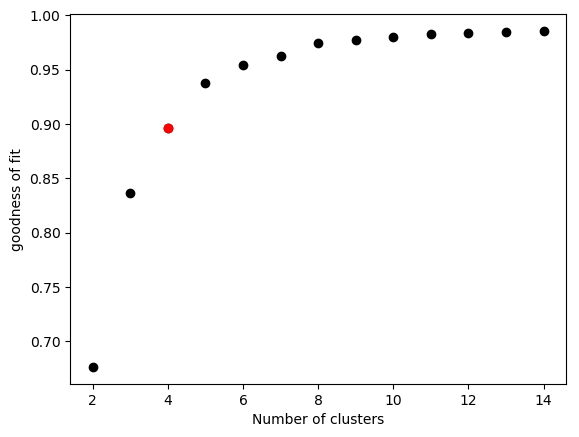
**

***Fig. S1***

*Goodness of fit (GVF) index for Jenks natural breaks algorithm on the full dataset*

*of primate group sizes.*

**Analysis of Sub-Orders**

We ran separate analyses at the species level for the two primate sub-orders. This yields an optimal division into four clusters with means at 4.63, 11.75, 25.99 and 53.71 (MLE) and 5.47, 16.28, 31.25 and 53.09 (Jenks) for haplorhines; for strepsirrhines, MLE yields just two clusters with means at 2.26 and 9.23, while Jenks yields an optimal division into three clusters with mean cluster sizes of 2.27, 6.84 and 14.95 (however, the number of species for the two larger clusters is very small) (see Fig. 1).

**Robustness of Clusters**

To determine how robust the clustering pattern is, we re-ran the analysis on genus mean group sizes and on a dataset listing the sizes of individual groups.

Mean group sizes for individual genera were calculated by averaging the species mean group sizes given in Table S1. MLE yielded an optimal division into three clusters, while the Jenks algorithm gave four clusters, at the following clusters mean values:

MLE compound Poisson: 3.36 13.54 36.99

Jenks: 4.20 15.53 30.49 55.87

These are clearly in agreement with each other, and in broad agreement with those obtained from the species-level analysis (Fig. 1).

We ran the analysis on an extended dataset of individual primate group sizes provided by DeCasien et al. (2017). The database consists of censuses of 936 individual groups distributed among 143 species.

It needs to be noted that there are a number of problems with this dataset. First, it is incomplete. For example, only 66 groups are listed for the genus *Papio*, yet a comprehensive dataset of all censused groups for this genus yields 422 groups (16). Second, the distribution of data among the species represented is far from even: the mean number of groups sampled per species is 6.55±8.88SD (range 1-46), with the distribution being highly right-skewed; 87 (60.3%) of the species are represented by <5 groups while 12 species are represented by >20 (and, in two cases, by as many as 46) groups. The frequency of species with large samples is likely to introduce significant sampling bias into the overall distribution. Third, several species (*Theropithecus,* *Rhinopithecus*, *Mandrillus*, *Pongo*) are represented by foraging groups rather than social groups. The first three of these genera sometimes form extremely large foraging groups – almost an order of magnitude larger than the known social group size in at least one case (MacCarron et al. 2016). In other cases (e.g. *Papio papio*), the listed mean group size is significantly inflated by an extremely large but atypical group in a very small sample (N=3, when in fact 32 groups are available in the literature: Dunbar et al. 2018).

The distribution of group sizes for any primate species is always likely to be right-skewed, in some cases extremely so: this is an inevitable consequence of the fact that, as with all mammals, primate group sizes are the outcome of a complex trade off between the costs and benefits of living in groups of a given size, the local environmental conditions (in terms of both predation risk and foraging conditions) and the effect that social viscosity has on fission rates (Dunbar et al. 2009; Dunbar & Sosis 2018). Natural growth rates will tend to result in some groups growing to large sizes (a soft upper limit), whereas predation risk will usually set a hard lower limit (Dunbar et al. 2009). The presence of even a very small number of unusually large groups will heavily influence the clustering algorithm as it tries to fit the dataset into a limited number of groupings.

Notwithstanding these concerns, this database does provide a large sample across a fairly comprehensive range of primate species and we therefore ran the analysis on it. The resulting mean cluster sizes were:

MLE compound Poisson: 5.18  17.22   38.28  112.55

Jenks: 9.62 34.91 87.77 212.40 

Four points may be noted. First, both methods find an optimal division into four clusters. Second, we see the signatures for the same numbers as in the analysis of species mean group sizes, although the two methods don't necessarily pick up the same numbers: there is a suggestion that one method divides two modes where another combines them (~5 and ~17 in MLE vs ~9.5 in Jenks; ~88 and ~212 in Jenks vs 112 in MLE). Third, there is a strong suggestion that the largest cluster is driven by the long right tail in the distribution: the result is a cluster with a very large mean that actually contains only a handful of groups. The Jenks cluster of 212, for example, contains just 5 of the 936 groups sampled (0.5%). Indeed, in the entire sample there are only 13 groups >100 (just 1.3%).

Since groups of >100 are extremely rare in primates, we excluded groups with N>100, and re-ran the analyses with a reduced sample of 923 groups. With four clusters again being optimal, the cluster mean values are:

MLE Compound Poisson: 4.78  14.80  29.62  56.35

Jenks: 5.89 19.61 39.09 75.93

These clusters are virtually identical to those obtained with the main dataset (i.e. species mean group sizes).

The bottom line is that these data reveal essentially the same pattern as the main analysis, but with some artificiality introduced in the upper values due to a handful of cases where either (a) individual groups have grown to very large sizes that are atypical for their species or (b) foraging groups rather than social groups have been included in the sample.

**Number of Adult Females**

The mean number of adult females per group is given in *ESM Dataset S1*. The clustering algorithms were unable to detect any consistent clusters in the distribution of female cohort size, and typically behaved very unstably. Instead, the number of adult females seems to be a simple correlate of social group size (Fig. S2). The least squares regression line has a slope of b=0.37.


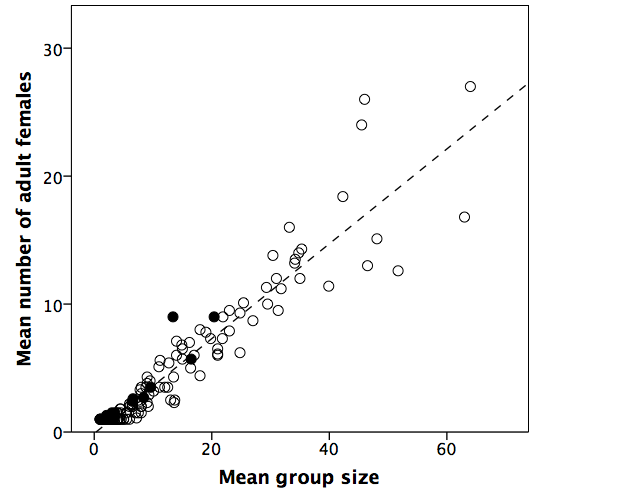


***Fig. S2***

*Mean number of adult females per group plotted against mean group size for individual species.*

**Social grades and rate changes**

The phylogenetic rate changes shown in Fig. 2 were classified into four categories using the *k*-*means* *clustering* algorithm in SPSS, and cross-tabulated with the clusters for group size identified in Fig. 1 (Table S1). In each case, the four clusters represent a natural monotonic progression (1 is smallest, 4 is largest). The cluster classifications for each species are given in *ESM Dataset S2.*

*Table S1. Cross-tabulation of species allocations to group size clusters*

*(from Fig. 1) and clusters in rate change (from Fig. 2)*

Rate change cluster

Group size cluster 1 2 3 4 Total

1 94 6 0 0 100

2 21 19 0 1 41

3 2 17 3 2 24

4 0 1 1 3 5

Total 117 43 4 6 170

Source of data: ESM Dataset S2

The distribution is significantly non-random (χ^2^=139.7, df=9, p<<0.0001) and there is a significant positive correlation between the two classifications (adjusted for ties, Kendall’s τ=0.685, N=170, p<<0.0001).

**References**

Abernethy KA, White LJ, Wickings EJ (2002). Hordes of mandrills (*Mandrillus sphinx*): extreme group size and seasonal male presence. *Journal of Zoology*, 258, 131-137.

Aureli F, Schaffner CM, Boesch C, Bearder SK, Call J, Chapman CA, Connor R, Fiore AD, Dunbar RIM, Henzi SP, Holekamp K (2008). Fission-fusion dynamics: new research frameworks. *Current Anthropology*, 49, 627-654.

Bettridge C, Lehmann J, Dunbar RIM (2010). Trade-offs between time, predation risk and life history, and their implications for biogeography: a systems modelling approach with a primate case study. *Ecological Modelling*, 221, 777-790.

Campbell CJ, Fuentes A, Mackinnon KC, Panger M, Bearder SK (eds) (2007). *Primates in Perspective*. Oxford University Press.

Clauset A, Shalizi CR, Newman MEJ (2009). Power-law distributions in empirical data. *SIAM Review*, 51, 661-703.

Coulson MR (1987). In the matter of class intervals for choropleth maps: with particular reference to the work of George F. Jenks. *Cartographica: The International Journal for Geographic Information and Geovisualization*, 24, 16-39.

DeCasien AR, Williams SA, Higham JP (2017). Primate brain size is predicted by diet but not sociality. *Nature Ecology and Evolution*, 1, 0112.

Dunbar RIM (2011). Evolutionary basis of the social brain. In: Decety J, Cacioppo J (eds) *Oxford Handbook of Social Neuroscience*, pp. 28-38. Oxford University Press.

Dunbar RIM, MacCarron P (in press). Fertility and predation risk as determinants of group sizes and social sturcture in baboons. *Journal of Human Evolution*.

Dunbar RIM, Sosis R (2017). Optimising human community sizes. *Human Behavior and Evolution*, 39, 106-111.

Dunbar RIM, Korstjens AH, Lehmann J (2009). Time as an ecological constraint. *Biology Reviews*, 84, 413-429.

Dunbar RIM, Arnaboldi V, Conti M, Passarella A (2015). The structure of online social networks mirrors those in the offline world. *Social Networks*, 43, 39-47.

Dunbar RIM, Cheyne S, Lan D, Korstjens AH, Lehmann J, Cowlishaw G (submitted). A time budgets model of gibbon socioecology.

Hill RA, Lycett JE, Dunbar RIM (2000). Ecological determinants of birth intervals in baboons. *Behavioral Ecology*, 11, 560-564.

Hongo S (2014). New evidence from observations of progressions of mandrills (*Mandrillus sphinx*): a multilevel or non-nested society? *Primates*, 55, 473-81.

Jarman PJ (1974). The social organization of antelope in relation to their ecology. *Behaviour*, 48, 215-268.

Jenks GF (1967). The data model concept in statistical mapping. *International Yearbook of Cartography*, 7, 186-190.

Jovani R, Mavor R, Oro D. (2008). Hidden patterns of colony size variation in seabirds: a logarithmic point of view. Oikos, 117, 1774e1781.

Jovani R, Mavor R. (2011). Group size versus individual group size frequency distributions: a nontrivial distinction. *Animal Behaviour*, 82, 1027-1036.

Kappeler PM (1997). Determinants of primate social organization: comparative evidence and new insights from Malagasy lemurs. *Biological Reviews*, 72, 111-151.

Kordsmeyer T, MacCarron P, Dunbar RIM (2017). Sizes of permanent campsites reflect constraints on natural human communities. *Current Anthropology*, 58, 289-294.

Lehmann J, Korstjens AH, Dunbar, RIM (2008) Time and distribution: a model of ape biogeography. *Ethology, Ecology and Evolution*, 20, 337-359.

Lehmann J, Korstjens AH, Dunbar RIM (2010). Apes in a changing world – the effects of global warming on the behaviour and distribution of African apes. *Journal of Biogeography*, 37, 2217-2231.

MacCarron P, Dunbar RIM (2016) Identifying natural grouping structure in gelada baboons: a network approach. *Animal Behaviour*, 114, 119-128.

MacCarron P, Kaski K, Dunbar RIM (2016). Calling Dunbar’s numbers. *Social Networks*, 47, 151–155.

Mackinnon J (1974). The behaviour and ecology of wild orang-utans (*Pongo pygmaeus*). *Animal Behaviour*, 22, 3-74.

Singleton I, van Schaik CP (2001). Orangutan home range size and its determinants in a Sumatran swamp forest. *International Journal of Primatology*, 22, 877-911.

Smuts BB, Cheney DL, Seyfarth RL, Struhsaker TT, Wrangham RW (eds) (1987). *Primate Societies*. Chicago University Press.
